# Supplementary material for: Analysis and optimization of fire evacuation safety performance in large urban complexes
Source: PLoS One. 2025 Nov 17;20(11):e0336964. doi: 10.1371/journal.pone.0336964 (PMC12622778; doi:10.1371/journal.pone.0336964)
Supplement: Attachment — Submitted filename: Response to Reviewers.docx [file pone.0336964.s002.docx]

**Dear Editor:**

Manuscript ID PONE-D-25-24987 entitled " Analysis and Optimization of Fire Evacuation Safety Performance in Large Urban Complexes " which has been revised.

We are truly grateful to yours and the reviewers’ critical comments and thoughtful suggestions. Based on these comments and suggestions, **we have made significant modifications on the original manuscript**. All changes made to the text are in **red** color.

We hope the new manuscript will meet your magazine’s standard. Please do forward our heartfelt thanks to these experts. We look forward to hearing from you soon for a favorable decision.

We tried our best to improve the manuscript and made a significant change in the manuscript. Below you will find our point-by-point responses to the reviewers’ and editor’s comments/ questions.

Reviewers' comments:

**Reviewer #1:**

This paper presents a research on analysis and optimization of fire evacuation safety performance in large urban complexes based on the Building Information Modeling (BIM).

1. However, the description of the abstract needs to be redesigned.

**Reply**: Many thanks for this comment. After review and proofreading, we have comprehensively revised the introduction section in accordance with your suggestions, with improvements made to its logical structure. The narrative logic of the introduction has been reorganized, adopting the logical thread of "research background—existing problems—research objectives—research methods—case verification—research significance". The feasibility and effectiveness of the research have been demonstrated through specific methods and case data.

The following is the revised content:

“

Urban large-scale complexes, such as shopping malls, pose significant challenges for fire safety management due to their intricate spatial layouts, high population density, and diverse occupancy characteristics. Efficient fire evacuation strategies are critical to minimizing casualties and economic losses, yet existing approaches often overlook the dynamic interplay between fire propagation and human behavior, leading to suboptimal safety assessments. This study proposes an integrated simulation framework to optimize evacuation strategies by coupling fire dynamics with pedestrian flow modeling, aiming to enhance both evacuation efficiency and personnel safety. The methodology comprises three key steps: (1) Fire scenario simulation: A Building Information Modeling (BIM)-based digital platform is constructed to simulate fire propagation. Critical fire parameters (e.g., heat release rate, combustion model) are calibrated to quantify temporal variations in smoke temperature, CO concentration, and visibility across different zones. (2) Evacuation dynamics modeling: A pedestrian evacuation model is developed by integrating demographic factors (age structure, movement speed, population density) and fire-induced regional risks, enabling realistic simulation of crowd movement under fire conditions. (3) Safety performance evaluation and strategy optimization: Safety margins at staircases are assessed by comparing Required Safe Egress Time (RSET) and Available Safe Egress Time (ASET), followed by a safety grading system to identify high-risk bottlenecks. Evacuation strategies are then optimized to mitigate these risks. A case study was conducted on a shopping mall in Chengdu to validate the framework. Simulation results indicate an initial evacuation time of 260.4 seconds. Safety performance analysis revealed critical risks at staircases A and C (1st floor) and D (2nd floor) due to insufficient safety margins. After strategy optimization, the total evacuation time was reduced to 245.5 seconds, with safety margins at the three high-risk staircases increased by 130.8 s, 115.2 s, and 72 s, respectively, fully meeting safety requirements. The overall evacuation efficiency was significantly improved. This study demonstrates the effectiveness of the proposed framework in quantifying fire risks and optimizing evacuation strategies for large-scale complexes. The integrated simulation approach provides a scientific basis for evidence-based safety management and evacuation planning, offering valuable insights for urban fire safety engineering and emergency response optimization.

”

2. What is the most important innovation point in this paper?

**Reply**: Many thanks for this comment. We have re-refined and sorted out the innovation points of this paper. The most important innovation of this study is: proposing an integrated simulation framework, which obtains a personnel evacuation model under the influence of fire by coupling fire dynamics with pedestrian flow modeling. Taking this model as the data basis, safety performance evaluation is carried out and evacuation strategies are optimized, aiming to improve both evacuation efficiency and personnel safety. In addition, simulations based on real scenarios are conducted to demonstrate the effectiveness and applicability of this method. We further highlight the innovation in the revised manuscript.

3. The description of data analysis is not detailed enough.

**Reply**: Many thanks for this comment. We have supplemented the insufficiently detailed parts in the data analysis. These include: the variation patterns of visibility across different floors in the fire simulation results and their causes; in the pedestrian evacuation simulation, further statistics and analysis on personnel's exit choices. By comparing the pedestrian flow rates and usage times among different exits, a more detailed personnel evacuation scenario has been constructed. Additionally, the safety of personnel's exit choices has been incorporated into the optimization analysis of personnel evacuation to enhance the overall safety and rationality of the optimization scheme.

The following is the revised section:

“

Figure 6 Simulation results of visibility variation across floors in the shopping center

Visibility is generally defined as the maximum distance at which a person can clearly see an object. The visibility conditions on each floor are illustrated in Figure 6. When a fire breaks out, a large amount of smoke is generated and quickly spreads to the surrounding rooms. By 142.5 seconds, the visibility in the first-floor restaurant drops below 10 meters, showing obvious horizontal diffusion characteristics. After 374.7 seconds of the fire, the smoke spreads to the vicinity of staircases C and D, and the vertical upward spread of the smoke causes the visibility on the second floor to gradually drop below 10 meters, with the smoke continuing to spread to upper floors. After 571.3 seconds of the fire, due to the deposition of smoke particles under the action of gravity, the visibility in most areas of the first and second floors continues to decrease. The corridor areas where staircases A and B are located have narrow spaces and poor ventilation conditions, leading to severe smoke accumulation and a rapid drop in visibility to below 10 meters. As for staircases C and D, their closer distance to the fire source results in longer smoke accumulation time, causing their visibility to drop below 10 meters. At the end of the 600-second simulation, the visibility in most positions on the first and second floors is below 10 meters, with only the areas near staircases E and G being less affected in terms of visibility. On the third floor, the visibility at staircases A, B, C, and the lobby near staircase D drops below 10 meters, while the visibility in other areas remains good.

Figure 9. Curve diagram of the variation of pedestrian flow rate with time at each exit of a shopping mall

The variation of pedestrian flow rate at each exit with evacuation time is shown in Figure 9. During the evacuation process, people tend to choose the nearest exit as their target. At 50 seconds after the start of evacuation, the pedestrian flow rate at each exit reaches its peak: the flow rate at Exit 1 is the highest, reaching 6.17 people/s; the flow rates at Exit 2 and Exit 5 reach 3 people/s; and the maximum flow rates at the remaining exits are around 2 people/s. Afterwards, the flow rates gradually decrease until 110 seconds into the evacuation, when the pedestrian flow rate at each exit stabilizes at approximately 1 people/s. As indicated in the table, without evacuation guidance, although the pedestrian flow rates at each exit stabilize after a certain period, the distribution of people is uneven and fails to avoid fire-affected areas, resulting in reduced evacuation efficiency and a narrowed safety margin.

Figure 16. Curve diagram of the variation of pedestrian flow rate with time at each exit after optimization of the personnel evacuation scheme

As can be seen from Figure 16, after the optimization of the personnel evacuation scheme, the pedestrian flow rates at exits with larger safety margins, such as Exit 1, Exit 2, and Exit 6, have significantly increased compared to those before optimization. The maximum pedestrian flow rate at Exit 2 reaches 4 people/s and remains above 2 people/s throughout the process, while that at Exit 6 stays above 1.5 people/s. This indicates an improved utilization rate of these exits, an accelerated personnel evacuation speed, reduced crowd congestion, and enhanced evacuation safety.

For exits in areas more affected by the fire with lower safety margins, including Exit 3, Exit 5, and Exit 7, the number of evacuees has been reduced by guiding people to other exits, thereby avoiding congestion caused by excessive crowd gathering. Specifically, the required safe evacuation time at Exit 5 has decreased from 260.53 s to 217.58 s, and that at Exit 7 has dropped from 233.63 s to 134.74 s. In contrast, the required safe evacuation time at Exit 3 has changed slightly, with a reduction of only 4.15 s, which helps improve its safety margin. As for Exit 4, due to the small number of people passing through, there is little difference in its performance before and after the optimization.

”

4. It is noted that your manuscript needs careful editing by someone with expertise in technical English editing paying particular attention to English grammar, spelling, and sentence structure so that the goals and results of the study are clear to the reader.

**Reply**: Many thanks for this comment. After our re-examination and proofreading, targeting the issues of grammar, tense, sentence structure, and spelling in the original text, we have corrected the incorrect spellings and sentence structures, unified the tenses, and improved the grammar to make it more consistent with the usage conventions of academic English.

5. Figures 6, 9, and 10 are not clear.

**Reply**: Many thanks for this comment. We have redrawn the aforementioned images with insufficient clarity, increased the text size in the images, and improved their quality and resolution. Figures 7, 11, and 12 in the revised manuscript correspond to Figures 6, 9, and 10 in the original manuscript.

The redrawn picture is as follows:

Figure 7. Curve of the Number of Evacuees on Each Floor of a Shopping Mall Changing with Time

Figure 11. Simulation Results of Visibility Changes at Stairway Entrances on Each Floor

Figure 12. Usage of Each Staircase for Personnel Evacuation

6. It lacks grid independence verification in numerical simulation calculations.

**Reply**: Many thanks for this comment. In the section "Fire Simulation Parameter Settings", we have added content related to grid independence verification. Specifically, by comparing the variation trends of fire products near the fire source under different grid size conditions, a consistency test was conducted to analyze the degree of differences, thereby performing the grid independence verification.

The following is the revised section:

“……

To verify grid independence, the following criteria are typically required:

1. Grid Convergence: When calculations are performed using different grids, the results should converge to similar solutions under sufficiently small grid sizes. As the grid size decreases, the computational results should gradually approach a stable solution.

2. Grid Independence: When calculations are conducted using different grids, the results should remain consistent under the same physical conditions. Even with varying grid sizes, the computational results should exhibit the same physical trends and characteristics.

3. Grid Smoothness: When calculations are carried out using different grids, the results should remain smooth under the same physical conditions. Even as the grid size changes, the computational results should not display significant isolated or singular points.

Therefore, in this paper, three groups of progressively decreasing grid sizes (1.2 m × 1.2 m × 1.2 m, 0.6 m × 0.6 m × 0.6 m, and 0.2 m × 0.2 m × 0.2 m) were set, and the variations in key indicators such as temperature, visibility, and CO concentration distribution near the fire source were recorded. By comparing the differences in the variation curves of the yield of the same fire product under different grid scales and conducting a significance analysis of these differences, the grid independence is verified.

Figure 2 Variation of Fire Product Yields Under Different Grid Sizes

As indicated in Figure 2, and the variation trends are similar without significant divergence points. In the significance analysis of differences, all P-values are greater than 0.05, indicating no significant difference in the mean values of the data. Therefore, grid independence is verified.

……”

7. The description of material properties parameters in simulation calculations is incomplete.

**Reply**: Many thanks for this comment. We have supplemented the incomplete parts of the material property parameters in the simulation calculations. The supplemented material coefficients include material type, density, specific heat, electrical conductivity, emissivity, absorption coefficient.

The following is the revised section:

“

The heat release rate growth coefficient depends on the type of combustible material, and different types of combustibles can lead to distinct types of fires. Shopping malls contain a large variety of combustible materials, such as wood, PVC, and foam materials that are widely used in decoration processes. In this paper, the types of combustible materials are set as wood, PVC, and foam materials, while the walls are set as concrete. The material parameters are presented in Table 1.

Table 1 Material Attribute Parameter Table

| Material Type | Density | Specific Heat | Conductivity | Emissivity | Absorption Coefficient |
| --- | --- | --- | --- | --- | --- |
| Wood | 640 kg/m³ | 2.85 kJ/(kg·K) | 0.14 W/(m·K) | 0.9 | 5.0E+4 1/m |
| PVC | 1380 kg/m³ | 1.44 kJ/(kg·K) | 0.16 W/(m·K) | 0.95 | 5.0E+4 1/m |
| Foam | 28 kg/m³ | 1.7 kJ/(kg·K) | 0.05 W/(m·K) | 0.9 | 5.0E+4 1/m |
| Concrete | 2280 kg/m³ | 1.04 kJ/(kg·K) | 1.8 W/(m·K) | 0.9 | 5.0E+4 1/m |

”

8. Figure 3 illustrates the temperature changes across different floors of the building. There has been no in-depth analysis of the reasons behind these phenomena.

**Reply**: Many thanks for this comment. We have re-analyzed this part and summarized the variation law of fire temperature across different floors, with supplementary content added to the original analysis.

The following is the revised section:

“

Figure 4 Simulation results of smoke temperature variation across floors in the shopping center

Figure 4 illustrates the temperature changes across different floors of the building. At 161 s after fire ignition, the smoke temperature in the first-floor restaurant reached 60°C. By the end of the simulation (600 s), only areas such as stairwells E and G on the first floor approached 60°C, while temperatures on other floors remained below this critical threshold.

As time elapses after ignition, due to the narrow space and limited airflow in the room where the fire source is located, heat accumulates more easily within the space, resulting in significant temperature changes. In contrast, in other areas, being relatively far from the fire source, and after fire smoke enters the hall, the internal space of the hall is larger, heat exchange is frequent, and the smoke dissipates more easily. These areas are significantly affected by the attenuation of thermal radiation and smoke energy transfer, leading to smaller temperature variations, all of which remain below 60°C.

”

9. The conclusion section is not clear enough, it is best to write three separate conclusions.

**Reply**: Many thanks for this comment. We have revised the conclusion section of the paper and summarized the experimental conclusions from three aspects .

The following is the revised content:

“

(1) Visibility has a more rapid and direct impact on the personnel evacuation process compared with smoke temperature and CO concentration. When smoke spreads, the decrease in visibility has an immediate impact on personnel evacuation. Although the increase in smoke temperature and CO concentration can also pose a serious threat to the personal safety of evacuees, it takes a certain amount of time for the increase in smoke temperature and CO concentration to reach a lethal level for the human body. Therefore, in the fire simulation process, the influence range and degree of visibility are greater than those of smoke temperature and CO concentration.

(2) By comparing the available safe evacuation time and required safe evacuation time for personnel evacuation at staircases across different floors, as well as the pedestrian flow rates and required safe evacuation time among various exits, the safety margins of each staircase were quantified. Furthermore, adjustments were made to personnel evacuation routes and directions based on the fire-affected area. This approach can more intuitively reflect the differences in safety performance among various components, thereby facilitating the proposal and implementation of reasonable optimization schemes. Focusing on staircases A, C, and D with potential safety risks in the shopping mall, as well as exits significantly affected by the fire, a comparison of the fire evacuation safety performance of the shopping mall before and after the optimization of fire protection design was conducted. Simulation results show that the safety margins of staircase A and C on the first floor, and staircase D on the second floor have increased by 130.8 s, 115.2 s, and 72 s, respectively. Additionally, the overall personnel evacuation time has been reduced by 14.9 s, the distribution of evacuees has been significantly optimized, and the overall evacuation safety has been improved.

(3) Based on fire evacuation simulation data and personnel evacuation simulation data, optimizing the personnel evacuation scheme can enhance the fire evacuation safety performance and improve the evacuation efficiency of the shopping mall. The research results of this paper can provide technical and methodological support for fire safety performance assessment and personnel evacuation scheme planning of large urban complexes.

”

**Reviewer #2:**

The main title is develop very well. The main idea is develop by chronological order. The main idea is develops very well. The main idea is discuss about STEM. The idea of BIM is develop very well. It should elaborate more.

**Reply**: Many thanks for this suggestion. Regarding the issue you raised that the explanation of the BIM concept is not detailed enough, we have added an elaboration on the BIM concept and its application in the field of fire protection.

The following is the revised content:

“

Building Information Modeling (BIM) is a digital technology-based tool for managing the entire life cycle of a building. Its core lies in constructing a 3D model that incorporates the building's physical properties, functional characteristics, and full-process data, thereby achieving information integration and collaborative management throughout the building's stages from planning and design, construction, operation and maintenance to even demolition. In the field of fire protection, BIM models can accurately integrate detailed information of the building's fire protection system, providing precise geometric and attribute data input for fire simulation software and evacuation simulation tools. This ensures that the simulation scenarios are highly consistent with the actual situation of the building, thereby enhancing the scientificity of fire risk assessment and evacuation strategy optimization.

”
