# Supplementary material for: Analysis and optimization of fire evacuation safety performance in large urban complexes
Source: PLoS One. 2025 Nov 17;20(11):e0336964. doi: 10.1371/journal.pone.0336964 (PMC12622778; doi:10.1371/journal.pone.0336964)
Supplement: Attachment — Submitted filename: Response_to_Reviewers_auresp_2.docx [file pone.0336964.s003.docx]

**Dear Editor:**

Manuscript ID PONE-D-25-24987R1 entitled "Analysis and Optimization of Fire Evacuation Safety Performance in Large Urban Complexes" which has been revised.

We are truly grateful to yours and the reviewers’ critical comments and thoughtful suggestions. Based on these comments and suggestions, **we have made significant modifications on the original manuscript**. All changes made to the text are in **red** color in the “Revised Manuscript with Track Changes” document.

We hope the new manuscript will meet your magazine’s standard. Please do forward our heartfelt thanks to these experts. We look forward to hearing from you soon for a favorable decision.

We tried our best to improve the manuscript and made a significant change in the manuscript. Below you will find our point-by-point responses to the reviewers’ and editor’s comments/ questions.

**#Reviewers' comments:**

The authors have thoroughly addressed the reviewers' concerns. The addition of grid independence verification, complete material property parameters, and enhanced analysis of evacuation dynamics significantly strengthen the manuscript. The integrated BIM-based framework provides practical value for fire safety assessment in large commercial complexes.

Minor Suggestions for Final Preparation

While I recommend acceptance, the authors may wish to address these minor points during final manuscript preparation:

1. Numerical Consistency: Please verify the evacuation time at Exit 5 (appears as both 260.4s and 260.53s in different sections). Also make the height unit consistent, choose m or cm.

**Reply**: Many thanks for this comment. Regarding the issue of inconsistencies in some experimental results, we re-examined the experimental results and unified the experimental conclusions across the study. Additionally, the unit for height was standardized to meters (m).

2. Statistical Test Specification: When stating "all P-values are greater than 0.05" for grid independence verification, please specify which statistical test was used.

**Reply**: Many thanks for this comment. To address the issue of ambiguity in the method used for verifying grid independence, we have further elaborated on the applied method—One-way Analysis of Variance (One-Way ANOVA)—and additionally included the calculation process to facilitate a more robust verification of grid independence.

The following is the revised section:

“One-way Analysis of Variance (One-Way ANOVA) is a statistical method used to determine whether there are significant differences in the means among multiple groups. Its core principle involves decomposing the total variation into between-group variation and within-group variation, and then using the F-test and P-value to judge the significance of between-group differences. This method is well-suited for the research design of this study, where "grid scale serves as the sole independent variable and key indicators act as continuous dependent variables". It complies with the assumptions of independence, normality, and homogeneity of variance for grid monitoring data, enabling quantitative verification of whether grid scale exerts a significant impact on key indicators and thus accurately aligning with the objective of verifying grid independence.

Therefore, this study set three groups of gradually reduced grid sizes (1.2m×1.2m×1.2m, 0.6m×0.6m×0.6m, and 0.2m×0.2m×0.2m). Taking visibility (V), CO concentration (CO), and temperature (T) near the fire source as the key observation indicators, a one-way ANOVA was conducted based on the continuous monitoring data obtained 600 seconds after the fire ignition (with a sample size of 1001 for each group). The differences in the variation curves of the same fire product yield under different grid scales were compared to verify grid independence. The variation of fire product yields under different grid sizes is illustrated in Figure 2, and the results of the one-way ANOVA are presented in Table 2.

Table 2. One-way ANOVA Results Table of Key Indicators Under Different Grid Scales

| Analytical Indicator | Grid Scale | Group Mean (± Standard Deviation) | F-statistic | P-value | Levene's Test | Conclusion (α=0.05) |
| --- | --- | --- | --- | --- | --- | --- |
| Visibility (V) | 1.2m | 10.23±12.65 | 1.555 | 0.211 | F=1.124 P=0.326 | Homogeneous variance;  No significant difference between groups |
|  | 0.6m | 10.87±12.71 |  |  |  |  |
|  | 0.2m | 11.02±12.83 |  |  |  |  |
| CO Concentration（CO） | 1.2m | 0.008±0.003 | 1.624 | 0.197 | F=0.987 P=0.373 | Homogeneous variance;  No significant difference between groups |
|  | 0.6m | 0.007±0.002 |  |  |  |  |
|  | 0.2m | 0.007±0.002 |  |  |  |  |
| Temperature (T) | 1.2m | 125.68±89.32 | 0.482 | 0.618 | F=1.352 P=0.259 | Homogeneous variance;  No significant difference between groups |
|  | 0.6m | 126.15±88.97 |  |  |  |  |
|  | 0.2m | 127.03±89.15 |  |  |  |  |

As shown in Figure 2, the variation trends of different fire product yields with fire development are similar under different grids and there are no significant divergence points. As presented in Table 2, in the results of one - way analysis of variance, among the yields of each fire product under different grid scales, the F - statistics of all indicators are relatively small, and all P - values are greater than 0.05, which meets the homogeneity of variance test. This indicates that changes in grid size will not significantly alter the results of key indicators such as visibility, CO concentration, and temperature, verifying that grid independence holds.”

3. Figure 2: Consider adding brief labels to panels (a), (b), (c) to indicate which parameter each represents.

**Reply**: Many thanks for this comment. Regarding the issue that the subplots in Figure 2 lack labels, we have divided the subplots and added standardized labels to facilitate better differentiation of different parameters.

The revised figure 2 is as following:

4. Minor Typographical: "be-tween" appears with incorrect hyphenation in one location.

**Reply**: Many thanks for this comment. Regarding the issues of incorrect punctuation usage in the manuscript, we have conducted a comprehensive check and revised all instances where punctuation was improperly used.
